# Altered microbiota, antimicrobial resistance genes, and functional enzyme profiles in the rumen of yak calves fed with milk replacer

**DOI:** 10.1128/spectrum.01314-23

**Published:** 2023-11-28

**Authors:** Yimin Zhuang, Wei Guo, Kai Cui, Yan Tu, Qiyu Diao, Naifeng Zhang, Yanliang Bi, Tao Ma

**Affiliations:** 1 Key Laboratory of Feed Biotechnology of the Ministry of Agriculture and Rural Affairs, Institute of Feed Research, Chinese Academy of Agricultural Sciences, Beijing, China; 2 Key Laboratory of Animal Genetics, Breeding and Reproduction in the Plateau Mountainous Region, Ministry of Education, Guizhou University, Guiyang, China; Jilin University, Changchun, China

**Keywords:** rumen, milk replacer, yak, microbiome, metagenomics, resistome

## Abstract

The use of milk replacer (MR) is a modern strategy to ensure the health and growth of neonatal ruminants. Some studies have confirmed that dietary factors have significant effects on ruminal antimicrobial resistance genes (ARGs), which, as a potential threat to livestock, have received more attention. However, the effect of different milk sources (milk or MR) on the rumen resistome of yaks is still largely unknown. Here, using metagenome technology, we investigated the occurrence of ruminal ARGs in yaks and their relationship with rumen bacteria in response to different milk sources. The metagenomic result showed that MR feeding significantly increased the abundance of *Prevotella* members compared to milk feeding. A total of 138 rumen resistome-conveying ARGs to 17 different classes of antimicrobials were detected, of which tetracycline resistance was the predominant. MR feeding also drove the changes in the rumen resistome, and the predicted bacterial origins of ARGs had complex and various manifestations. Moreover, enzymes of butyrate metabolism and carbohydrate-active enzymes encoding carbohydrate and cellulose degradation were increased. This study revealed that MR feeding increased the abundance of beneficial bacteria *Prevotella* members, changed the microbial resistome, and promoted the metabolic function of volatile fatty acid biosynthesis in the rumen of yaks, which could provide beneficial evidence for the regulation of the rumen microbiome and the intervention of antimicrobial resistance in ruminants by dietary or nutritional methods.

**IMPORTANCE:**

Yaks, as ruminants inhabiting high-altitude environments, possess a distinct rumen microbiome and are resistant to extreme living conditions. This study investigated the microbiota, resistome, and functional gene profiles in the rumen of yaks fed milk or milk replacer (MR), providing insights into the regulation of the rumen microbiome and the intervention of antimicrobial resistance in yaks through dietary methods. The abundance of *Prevotella* members increased significantly in response to MR. Tetracycline resistance was the most predominant. The rumen of yaks contained multiple antimicrobial resistance genes (ARGs) originating from different bacteria, which could be driven by MR, and these ARGs displayed intricate and complex interactions. MR also induced changes in functional genes. The enzymes associated with fiber degradation and butyrate metabolism were activated and showed close correlations with *Prevotella* members and butyrate concentration. This study allows us to deeply understand the ruminal microbiome and ARGs of yaks and their relationship with rumen bacteria in response to different milk sources.

## INTRODUCTION

The yak (*Bos grunniens*) is a major ruminant residing on the Qinghai-Tibet Plateau that shows strong resistance to harsh environments (1). Local herdsmen have been raising yaks for meat, milk, and combustible feces for generations (2). Yak calves are weaned naturally or artificially at 1.5–2 years old under extensive conditions (3). Suckling has been shown to delay the resumption of estrous cycling in postpartum cows (4, 5), which may lead to the poor reproductive performance (calving every 2–3 years) of female yaks. On the other hand, early weaning based on feeding commercial milk replacer (MR) has been reported to meet the nutrition requirements of neonatal ruminants as well as shorten the breeding interval of dams (6, 7), which could be potentially applied to shorten the weaning period of yak calves as well as the breeding period of yaks.

The rumen is a critical organ for the digestion and metabolism of nutrients in ruminants. Volatile fatty acid (VFA) produced by rumen microbial fermentation is the main energy source for ruminants, which provides more than 75% of the metabolic energy (8, 9). In preweaning calves, the intervention of appropriate dietary regimes can promote the development of the rumen microbiota as well as fermentation capacity (10, 11). A recent study showed that feeding MR increased the relative abundance of *Ruminococcus* (12), a genus that contributes to the degradation of cellulose and starch (13). Another study showed that, compared with waste milk, calves fed MR had a greater concentration of total volatile fatty acids in their rumen at 2 months old (14). The conversion of pyruvate to individual VFA is driven by a carbohydrate-active enzyme (CAZyme) produced by the rumen microbiota (15). However, compared with commercial herds, yaks have a unique rumen microbial community (16, 17), and the profiles of CAZyme in the rumen of yak calves remain largely unclear.

Antimicrobial resistance (AMR) has been posing huge threats to global public health, which reduces the therapeutic efficacy of antibiotics and increases the death rate of humans as well as food-producing animals (18, 19). Antimicrobial resistance genes (ARGs) can be transmitted horizontally between farm animal gastrointestinal microbial communities (20) or spread to humans via food and the environment. Recent studies have shown that the rumen of ruminants harbors a vast reservoir of ARGs, or resistomes (21), and revealed that the profiles and abundance of resistomes could be affected by diet (22) and/or antibiotic treatment (23). However, there is a lack of knowledge of the resistome profiles in the gastrointestinal tract of ruminants living under extreme conditions (such as yaks).

The objective of this study was to determine how MR feeding affects the rumen microbiome of yak calves. We hypothesized that feeding MR could enhance rumen fermentation in yak calves, which may be associated with changes in profiles of bacterial composition, functional genes, and rumen resistome.

## RESULTS

### Effect of MR feeding on diversity and composition of the yak rumen microbiota

A total of 1,110,803,772 reads from 15 rumen fluid samples of yak calves were generated by metagenomic sequencing [a sample in the treatment (TRT) group failed to pass library construction and was excluded], with an average of 74,053,584 ± 1,409,025 (mean ± standard deviation) reads per sample. After quality control (QC) and removing host genes, reads were annotated in the National Center for Biotechnology Information (NCBI) nr database. The rumen microbiome of yak calves consisted of 98.67 ± 0.21% bacteria, 1.06 ± 0.20% eukaryotes, 0.21 ± 0.02% archaea, and 0.02 ± 0.00% viruses.

**FIG 1:**
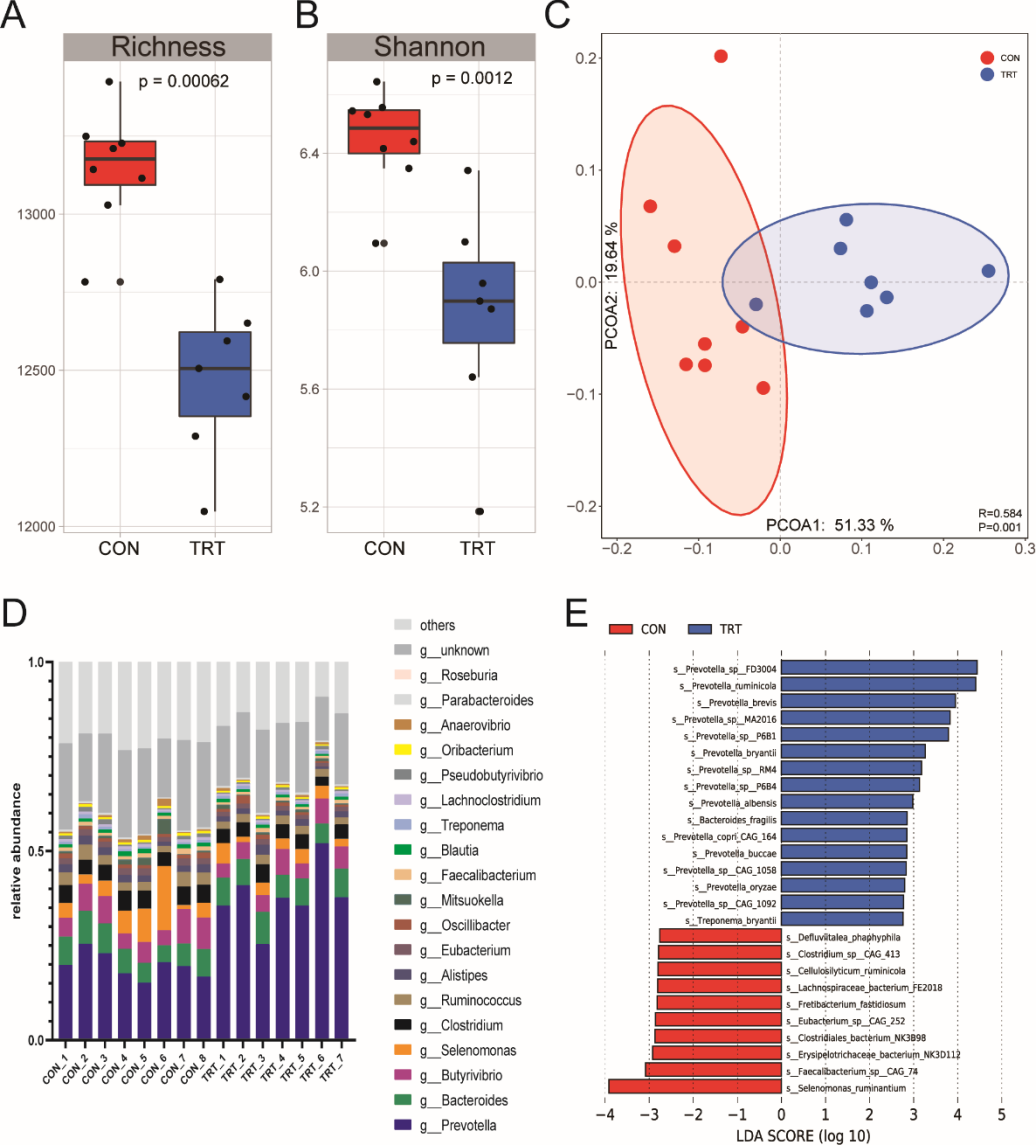
Microbial diversities and structure of rumen in yak claves. (**A and B**) The richness and Shannon index of the rumen microbiome in the CON and TRT groups. (**C**) The PCoA of the Bray-Curtis distances of the rumen microbiome between the CON and TRT groups. (**D**) The composition of the rumen microbiome at the gene level in the CON and TRT groups. (E) The identification of signature species in the CON and TRT groups using linear discriminant analysis effect size.

The microbial richness (Chao1 index; *P* < 0.001) and diversity (Shannon index; *P* = 0.001) were lower in the TRT group than in the control (CON) group (Fig. 1A and B). The principal coordinate analysis (PCoA) plot based on Bray-Curtis distance showed clear separation of the rumen microbiota of yak calves between the CON and TRT groups [*P* < 0.001, analysis of similarity (ANOSIM) *R* = 0.584] (Fig. 1C).

Firmicutes and Bacteroidetes were the predominant bacterial phyla in all samples (Fig. S1). The relative abundance of Firmicutes and Proteobacteria was lower (*P* < 0.001), while that of Bacteroidetes (*P* < 0.001) was higher in the TRT group (Fig. S1B). At the genus level, the top 20 accounted for 82.4% of total abundance, with *Prevotella* being most abundant in all samples (Fig. 1D). The relative abundance of *Prevotella* (*P* < 0.001) was higher, while that of *Eubacterium* (*P* < 0.001), *Faecalibacterium* (*P* = 0.021), *Blautia* (*P* < 0.001), *Lachnoclostridium* (*P* = 0.009), and *Oribacterium* (*P* < 0.001) was lower in the TRT group (Fig. S2). Furthermore, linear discriminant analysis (LDA) effect size (LEfSe) was performed to identify the signature rumen microbiota in the two groups at the species level (Fig. 1E). Consistent with the genus-level results, in the TRT group, most signature species belonged to the genus *Prevotella*, including *P. ruminicola*, *P. brevis*, and *P. bryantii*. In addition, *Bacteroides fragilis* and *Treponema bryantii* were also signatures in the TRT group, while *Selenomonas ruminantium*, *Faecalibacterium* sp. CAG 74, and *Clostridiales bacterium* NK3B98 were signatures in the CON group.

### Effect of MR feeding on yak rumen resistome

**FIG 2:**
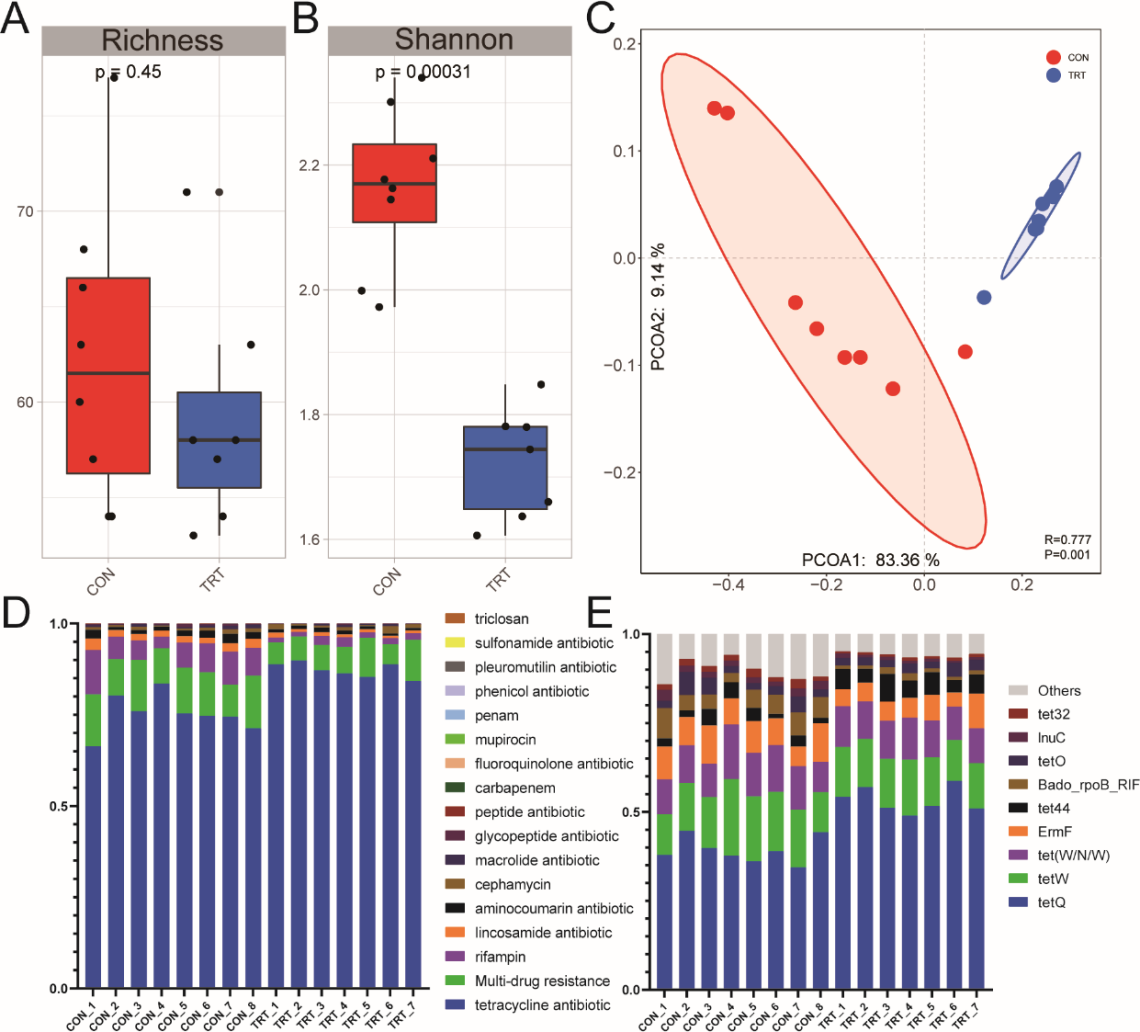
The rumen resistome structure and composition in the CON and TRT groups. (**A and B**) The richness and Shannon index of the rumen resistome in the CON and TRT groups. (**C**) PCoA of Bray-Curtis distances for the rumen resistome structure between the CON and TRT groups. (**D**) Relative abundance of ARGs by class of antibiotics per sample. (E) The composition of ARGs per sample.

The post-QC reads were assembled into 7,137,647 contigs. These contigs were annotated in the Comprehensive Antimicrobial Resistance Database, and 138 ARGs conferring resistance to 17 ARG classes were identified. There was no significant difference in the richness of observed ARGs between the two groups (Fig. 2A), while the diversity (Shannon index) in the TRT group was lower than that in the CON group (*P* < 0.001) (Fig. 2B). Moreover, according to PCoA analysis, we observed that the composition of ARGs showed a distinct difference between the two groups (*P* = 0.001, ANOSIM *R* = 0.777) (Fig. 2C).

**FIG 3:**
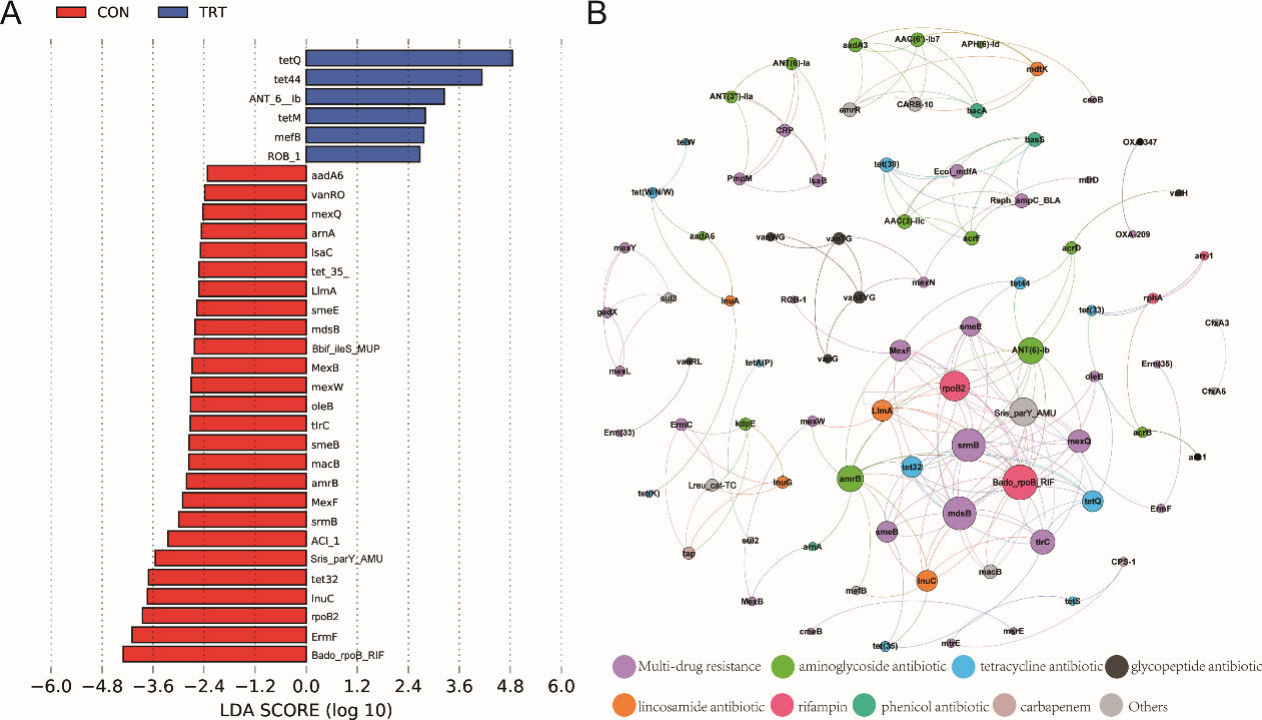
The identification of signature ARGs in the CON and TRT groups and their interactions. (**A**) The signature ARGs in the CON and TRT groups by the LEfSe algorithm. (**B**) Network analysis of the interactions among ARGs. The nodes (resistance genes) were colored by the corresponding class of antibiotics, and the size of the node was determined by the connection degrees.

Genes conferring resistance to tetracycline were dominant in both groups, accounting for over 60% of total abundance, followed by multidrug and rifampin classes (Fig. 2D). Specifically, compared with the CON group, tetracycline resistance showed a higher abundance in the TRT group. Conversely, multidrug resistance was higher in abundance in the CON group. For individual ARGs, *tetQ*, *tetW*, *tet* (*W/N/W*), and *ermF* were the most abundant ARGs across all samples (Fig. 2E). In the TRT group, six individual ARGs were identified as signatures, including *tetQ*, t*et44*, and t*etM*. More signature ARGs (24) were detected in the CON group, such as *mexQ*, *aadA6*, *ErmF*, *tet32*, and *Bado_rpoB_RIF* (Fig. 3A).

To better understand the interactions of ARGs in the rumen, the ARGs in the metagenomic sequenced rumen samples were applied to perform co-occurrence patterns according to the network inference model. The network revealed that the ARGs conferring resistance to multiple drugs, including *srmB*, *mexQ*, and *mdsB*, were the major nodes containing the wide connections. Of note, *Bado_rpoB_RIF* conferring resistance to rifampin was the most dominant node, which built a bridge to connect other ARGs (Fig. 3B).

### The prediction of the microbial origin of observed ARGs in the rumen

**FIG 4:**
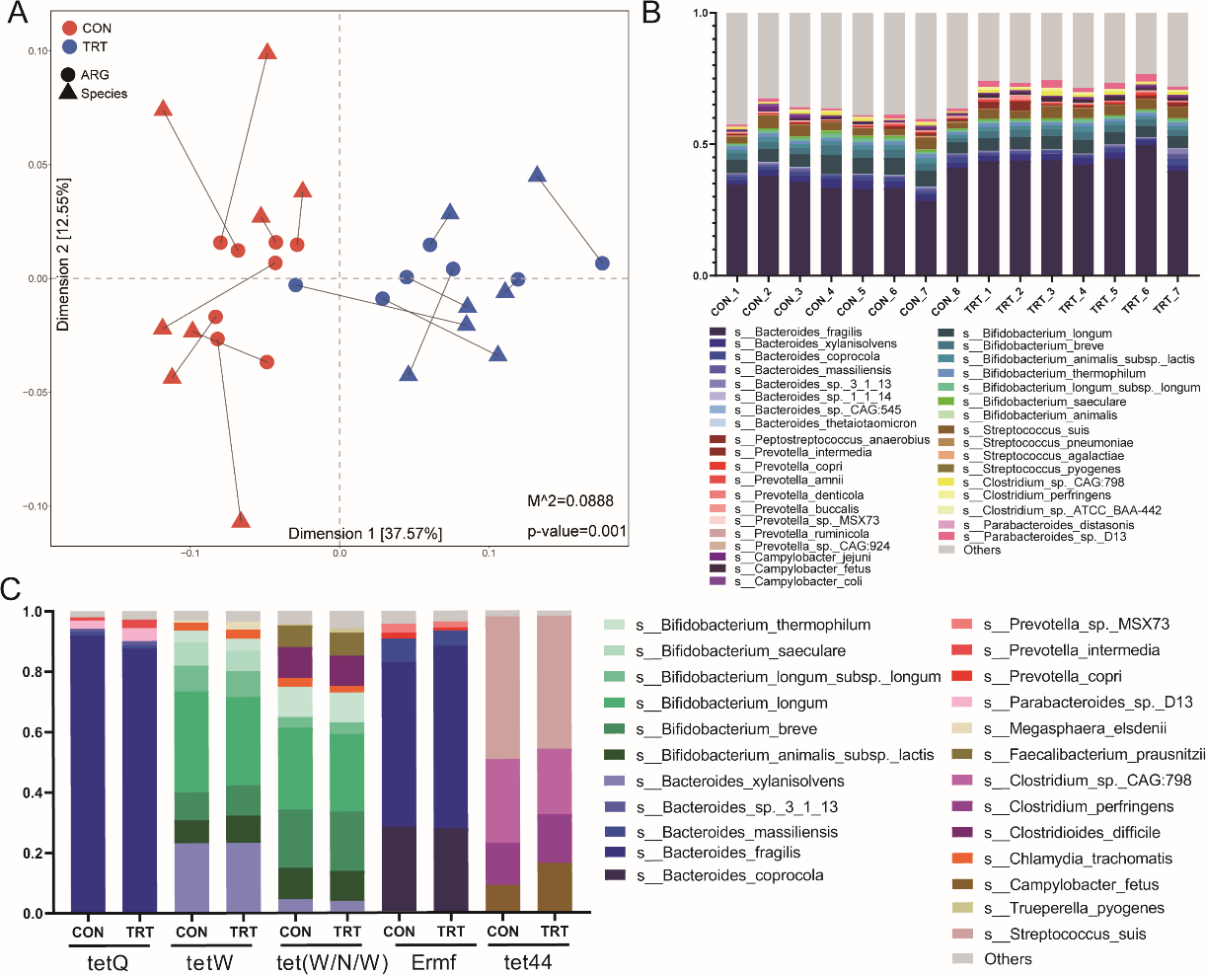
Rumen resistome is associated with its bacterial community. (**A**) Procrustes analysis of the association between the composition of the resistome and that of the bacterial community in the CON and TRT groups. (**B**) The proportion of whole ARGs annotated to the bacterial species. (**C**) The proportion of the top five most abundant ARGs annotated to the bacterial species.

First, a significant correlation between the composition of microbial communities and that of ARG profiles was confirmed by Procrustes analysis (*M* ^2^ = 0.0889 and *P* = 0.001) (Fig. 4A), suggesting that communities with similar microbial compositions had similar resistomes. Then, ARG-containing contigs generated by metagenomic assembly were used to predict the bacterial origin of ARGs. The bacterial genus of ARGs was mainly composed of the genera *Bacteroides*, *Bifidobacterium*, *Streptomyces*, and *Prevotella*. At the species level, the detected ARGs were predicted to belong to 1,159 different bacterial species across all the samples, with *Bacteroides fragilis*, *Bacteroides coprocola*, *Bacteroides xylanisolvens*, *Bifidobacterium longum*, *Bifidobacterium breve*, *Streptococcus suis*, and *Prevotella intermedia* representing over 60% of the total ARG abundance. Among them, *Bacteroides fragilis* was the most important ARG carrier in both groups and showed a higher abundance in the TRT group than in the CON group (Fig. 4B).

In addition, the bacterial species origins of the predominant ARGs (the top 5 ARGs represented) in the ruminal resistome of each group were predicted. As shown in Fig. 4C, the ARGs of *tetQ* and *ErmF* were mainly composed of *Bacteroides fragilis* and *Bacteroides coprocola*. The ARGs of *tetW* and *tet* (*W/N/W*) were mainly composed of *Bifidobacterium longum* and *Bifidobacterium breve*. The ARG *tet44* was mainly composed of *Clostridium perfringens*, *Clostridium* sp. CAG 798, and *Streptococcus suis*, which suggested that the different ARGs had distinguishable differences in bacterial origins.

### Effect of MR feeding on CAZyme profiles in yak rumen

**FIG 5:**
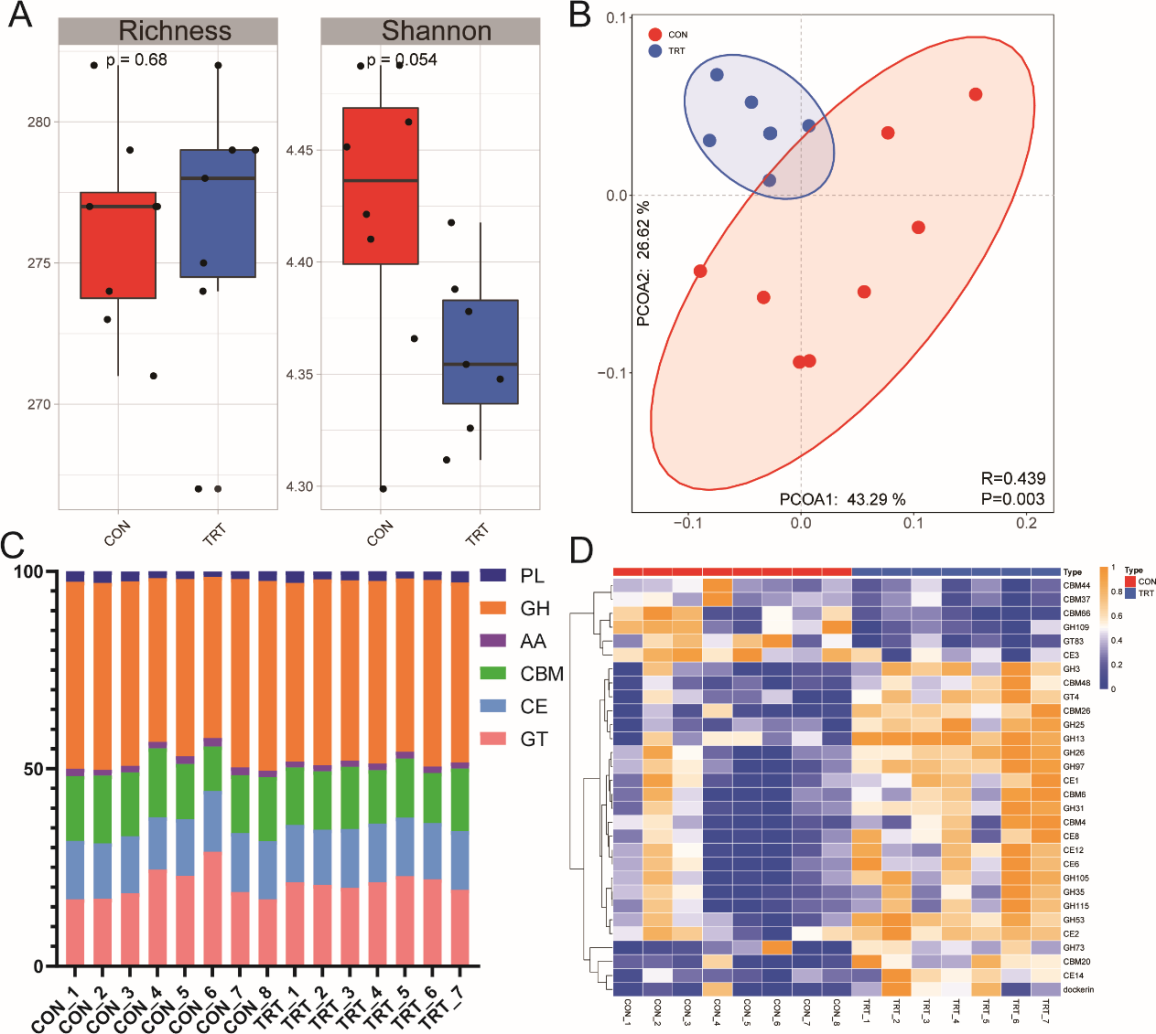
The structure and abundance of CAZyme in the CON and TRT groups. (**A**) The richness and diversity of rumen CAZyme in the CON and TRT groups. (**B**) PCoA of Bray-Curtis distances for the rumen CAZyme structure between the CON and TRT groups. (**C**) The bar plots of CAZyme at the class level in the CON and TRT groups. (**D**) The chord diagram shows the distribution of the top 30 CAZymes in the CON and TRT groups.

We also analyzed the CAZymes in the rumen microbiome to obtain insights into this important function for the growth and health of yak calves. The richness was not different between the two groups (*P* = 0.68), while the Shannon index in the TRT group tended to be lower than that in the CON group (*P* = 0.054) (Fig. 5A). A significant difference in the functional distribution of CAZymes was identified based on PCoA between the CON and TRT groups (*P* = 0.003, ANOSIM *R* = 0.439) (Fig. 5B). We further identified that the rumen microbiome of yak calves was affiliated with six major functional categories: glycoside hydrolases (GH), glycosyl transferases (GT), carbohydrate esterases (CE), carbohydrate-binding modules (CBM), auxiliary activities, and polysaccharide lyases. In all rumen microbial samples, over 60% of CAZyme genes were enriched in GT and GH (Fig. 5C).

Compared with the CON group, the relative abundance of GH (*P* = 0.46) was higher, while that of GT (*P* = 0.54) was lower in the TRT group (Fig. 5C). Differential abundance analysis showed that the relative abundance of 30 out of the top 50 CAZymes was significantly different between the CON and TRT groups (*P* < 0.05) (Fig. 5D). Compared to the CON group, most of these 30 CAZyme genes had a higher relative abundance (*P* < 0.05), and only a few CAZyme genes (CBM37, CBM44, CBM66, GH39, GH109, GT83, and CE3) had a lower relative abundance (*P* < 0.05) in the TRT group.

A network of co-occurrence patterns between CAZymes and bacteria showed that the relative abundance of *Prevotella* members (e.g., *P. ruminicola*, *P. bryantii*, and *P. brevis*), *Bacteroides* sp. CAG 1060, and *Butyrivibrio proteoclasticus* was positively correlated with the most CAZymes. On the contrary, the relative abundance of *Selenomonas ruminantium* had a negative correlation with GT27, CE3, and GH4. *Ruminococcus flavefaciens* was negatively correlated with GT92, GH23, and GT41 (Fig. S3).

### Effect of MR feeding on rumen fermentation and related metabolic pathways for carbohydrate degradation

**FIG 6:**
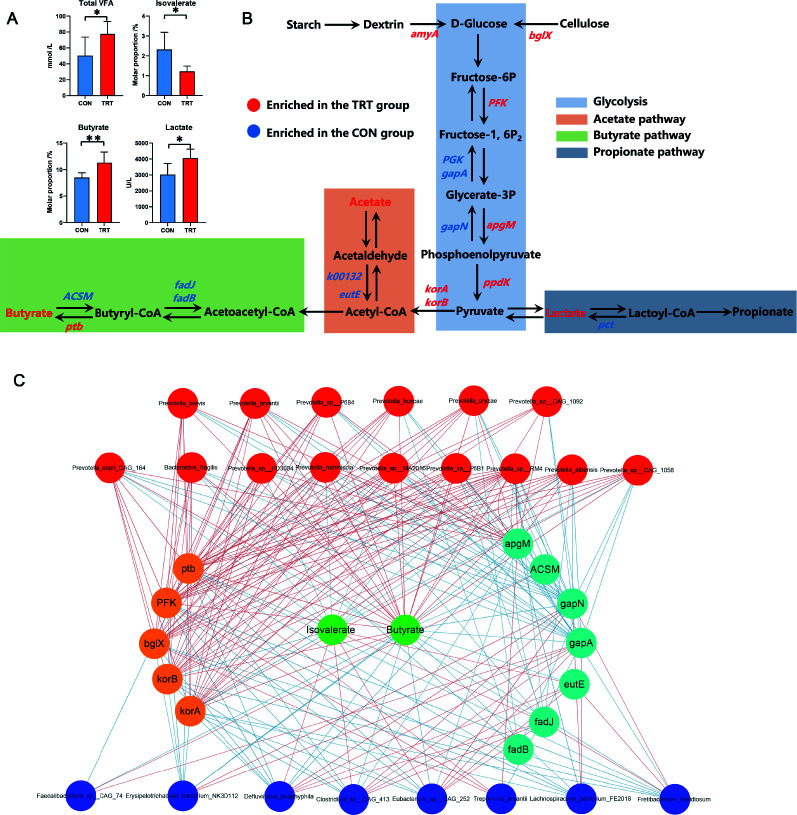
MR affected the rumen fermentation and related metabolism in the rumen microbiome, according to metagenomic results. (**A**) The comparison of VFA and lactate concentrations in the CON and TRT groups. (**B**) Comparisons of the relative abundance of related KO genes using the Wilcoxon test. The red font indicated that enzyme genes were significantly enriched in the CON group. The blue font indicated that enzyme genes were significantly enriched in the TRT group. (**C**) The tertiary network of bacteria, functional genes, and VFAs. Red nodes represent the signature species in the TRT group. Blue nodes represent the signature species in the CON group. Orange and light blue nodes represent the increased and decreased enzymes in the TRT group, respectively. Green nodes represent the VFAs. Red lines represent the positive correlations, and blue lines represent the negative correlations.

The rumen in ruminants digested the carbohydrates from feed efficiently via microbiota fermentation to obtain the energy required for growth and maintenance. In this study, compared with the CON group, we observed that the concentrations of total VFA and lactate and the relative abundance of butyrate were higher in the TRT group (*P* < 0.05) (Fig. 6A). According to the analysis of the metabolic pathway, the genes involved in starch and cellulose degradation were changed in response to MR feeding, including two upregulated genes, *amyA* and *bglX* (*P* < 0.05) (Fig. 6B). In the following glycolysis metabolism, the genes *PFK*, *apgM*, and *ppdK* showed higher abundance in the TRT group. In contrast, the genes *PGK*, *gapA*, and *gapN* showed higher abundance in the CON group (*P* < 0.05) (Fig. 6B). Similarly, for acetate and butyrate metabolism, the enzyme genes catalyzing the reaction, including *korA*, *korB*, and *ptb*, were higher in the TRT group. The enzyme genes reversing the catalyze reaction, including *k00132*, *eutE*, *fadJ*, *fadB*, and ACSM, were higher in the CON group, which was consistent with the higher concentrations of VFAs in the TRT group (*P* < 0.05) (Fig. 6B). In addition, we further constructed a tertiary network of bacteria, functional genes, and volatile acid metabolites (Fig. 6C). We observed that the signature taxa (identified by LEfSe) in the TRT group (mainly *Prevotella* members; red nodes) showed distinct correlations with the increased metabolic enzyme genes, including *ptb*, *PFK*, *bglX*, *korB*, and *korA*, which were also positively associated with butyrate. In contrast, the signature species (identified by LEfSe) in the CON group (blue nodes) showed negative relationships with the increased enzymes and butyrate.

## DISCUSSION

In this study, we used the metagenomic sequencing method to compare the effect of different liquid feeds (milk replacer vs dam’s milk) on the rumen microbiome of yak calves. A lower rumen microbial diversity was observed in yak calves fed MR than in those fed dam’s milk, which was in accordance with previous findings in lambs (25, 26). For young ruminants, most of the early colonized microbiome in the rumen is derived from their mother, including teats, feces, and milk (24, 27). In this study, the yaks in the TRT group were separated from the dams and consumed MR instead of milk, restricting vertical transmission of maternal microbiota, which might be an important reason for the lower microbial diversity. In addition, shifts in dietary regime could cause a severe stress response in ruminants with an immature gastrointestinal environment (28) and the impact on microbial diversity is inevitable.

Moreover, our results showed that at the age of 6 months, the rumen bacterial composition of MR-fed yaks was distinguished from that of yaks fed with milk, and *Prevotella*, attributed to the phylum Bacteroidetes, was the most dominant genus in both groups, and MR feeding increased its abundance further. *Prevotella ruminicola*, *Prevotella brevis*, and *Prevotella bryantii* were also signatures in the TRT group. *Prevotella* members are efficient utilizers of carbohydrates, including fiber and non-fiber (29, 30), and involved in the degradation of oligopeptides into amino acids (31). Therefore, in our study, we consider that the nutrient-rich and sufficient MR created a better material environment for *Prevotella* to proliferate, and *Prevotella* further yielded a higher level of VFA for rumen development and yaks’ growth. *Bacteroides* was the high-abundance genus following *Prevotella* across all the samples. Although *Bacteroides* showed the highest abundance in the rumen of newborns consuming milk and its abundance decreased with the introduction of solid feed, *Bacteroides* was still the long-term resident in the rumen due to its efficient degradation of saccharides from the diet (32). In addition, *Butyrivibrio* was the dominant bacteria in both groups of this study. *Butyrivibrio* was a primary VFA producer via degrading dietary fibers (33). Another study also proved that *Butyrivibrio* was identified as one of the main bacteria in the rumen of yaks from 4 to 6 months (34), which is consistent with our results.

In addition, we found that *Blautia and Faecalibacterium* showed a lower abundance in the TRT group than in the CON group, and LEfSe proved that *Faecalibacterium* sp. CAG 74 was the signature in the CON group. *Blautia* has been identified as a probiotic that has the ability to regulate host metabolism and maintain an environment in a steady state (35). *Faecalibacterium* has been proven to be closely related to resistance to intestinal inflammation in recent years. One study pointed out that *Faecalibacterium*, as a potential biomarker for the diagnosis of intestinal health, was reducing the abundance of intestinal disorders (36). Hence, in this study, rumen stress caused by weaning might disturb the balance of the microbiota and suppress the proliferation of these bacteria.

In our study, the ruminal microbiome of yak claves was also detected in 138 ARGs, conferring resistance to 17 ARG classes. Although all the yak calves did not receive any antibiotic therapy and their diet contained no antibiotics during the whole experimental period, our result revealed that the rumen might be an important source of ARGs. Previous studies have proven that the rumen microbiome is a natural reservoir of ARGs (37, 38), even if no antibiotics have been administered (22, 39). Recently, studies also showed that the prevalence of ARGs is not necessarily related to the use of antibiotics in animals, including pigs (40), dairy cows (39, 41), and chickens (42), which was consistent with our results. It is worth noting that, compared to commercial cattle farms at low elevations, the ARGs in yaks show lower diversity and abundance (43), which might be due to the low biomass in the high-altitude environment limiting the horizontal transfer of ARGs (43).

More specifically, resistance to tetracycline, rifampin, and multidrug antibiotics was dominant in the rumen of yaks. Tetracycline is a class of broad-spectrum antibiotics with a phenanthane mother nucleus produced by some *Streptomyces*. It can form a reversible combination with the bacterial core ribosomal 30 s subunit and inhibit protein synthesis to achieve an antibacterial effect (44). Xue et al. recently demonstrated that in 49 ruminal samples of dairy cows without antibiotic treatment, genes belonging to tetracycline resistance were the most abundant (39), which is similar to our results. Rifampin can strongly bind to the β subunit of DNA-dependent RNA polymerase, inhibit the synthesis of bacterial RNA, and prevent the enzyme from connecting with DNA, thus blocking the process of RNA transcription and stopping the synthesis of DNA and protein. However, high intensity of rifampin resistance is rarely detected in the rumen in other studies, which differed from this study result. One study reported that infants without antibiotic exposure still inherited most of the ARGs from their mothers, and breast milk may be an important medium. The phenomenon of vertical transmission of ARGs has also been confirmed in dairy cows (25). Therefore, we speculated that before our experiment began, some ARGs originated from the dams receiving rifampin exposure and had been transferred into the rumen of yak claves. We also observed that multidrug resistance was an important part of antibiotic resistance in our study. It is reported that due to the abuse of broad-spectrum antibiotics, some Gram-positive and Gram-negative bacteria processed multidrug resistance patterns, which made it difficult for traditional antibiotics to treat related infections. In order to avoid the spread of multidrug resistance genes in farm animals, we should be more deliberate in the selection and use of antibiotics. In addition, it is well known that antimicrobial peptides (AMPs) are considered excellent substitutions for traditional antibiotics, which have extensive applicability and highly effective bactericidal activity. The antimicrobial peptide database also indicated that 100 out of 112 AMPs in the human host have confirmed their antibacterial activities. However, considerable data showed that bacterial resistance to AMPs revealed complex cross-resistance to different types and mechanisms of action, which corresponds with our results. Considering more and more AMPs are used in animal production, peptide antibiotic resistance warrants our vigilance and further investigation.

On the other hand, dietary factors may significantly modify the resistome of ruminants (the collection of all ARGs). The previous research showed that concentrate-fed cattle may have a higher number and variety of ARG in the rumen (22). Non-medicated dietary supplements (vitamin D, etc.) promoted the reproduction of drug-resistant bacteria in the gut of dairy cows (45). The colostrum serving as a carrier for ARGs led to the distribution of antibacterial resistance in the newborns (43). In our study, we also found distinct resistance structures and different signature ARGs between the CON and TRT groups. This phenomenon might be directly related to the change in the relative abundance of some microbial taxa in response to MR feeding. One study showed that the presence of ARGs adheres directly to the bacterial phylogeny (46). In our study, we also observed the complex microbial origins of ARGs and a strong correlation between the rumen resistome and microbiome. For instance, in our study, *Bacteroides fragilis* was the signature species in the TRT group with higher abundance, and it was the main bacterial origin of *tetQ*. Therefore, compared with the CON group, *tetQ* became the signature ARG with higher abundance in the TRT group due to the higher abundance of *Bacteroides fragilis*. Taken together, we reckoned the difference in liquid diet changed the abundance of host bacteria of these ARGs and in turn affected the resistance dynamics, which made it more feasible for us to control the abundance and spread of ARGs by regulating the microbiome abundance in the future.

The rumen microbiome contained many genes of functional enzymes to cope with the complex digestive environments of the host (30, 47). In our study, based on the CAZyme database, significant alternations in CAZyme families of yak rumen caused by feeding MR were observed. In the TRT group, 30 CAZyme families were significantly changed, and most of these CAZyme families increased compared with the CON group. Many studies have shown that GH families are a category of enzymes that can hydrolyze glycosidic bonds connected with carbohydrates and have high substrate specificity (48, 49). GH13, as the main α-amylase (EC 3.2.1.1) family, has the ability to catalyze the hydrolysis of α-1,4-glucoside keys in starch and associated alpha-glucan, which is a highly efficient decomposer of starch (50, 51). For the GH families, CBM families are identified as contiguous acid sequences within carbohydrate-active enzymes with a discreet fold having carbohydrate-binding activity, including cellulose (52, 53), which differ from other non-catalytic sugar-binding proteins. In our study, we found that the CBM48 and CBM20 families increased significantly in the TRT group. These two families all belong to starch-binding domains, which have similar enzyme specificity and function (54). Particularly, CBM48 originates from the above-mentioned GH13 pullulanase subfamily (55). These evidences may mean that MR contained more carbohydrates and led to the active expression of starch and glycogen-degrading enzymes.

According to network analysis, we found that the *Prevotella* members (e.g., *P. ruminicola*, *P. bryantii*, and *P. brevis*) showed a positive association with the GH, CE, and CBM families. Among these families, GH8, GH51 (EC.3.2.1.4), CE1 (EC 3.1.1.73), CE2, CE6, and CE12 (EC.3.1.1.72) have been demonstrated to characterize cellulase (56) *Bacteroides* sp. CAG 1060, a degrader of saccharides (32), also had a correlation with CBMs. These results revealed the functional consistency between genes and bacteria. The synthesis of digestive enzymes depends on the coding genes carried by the related microorganisms. In our study, some CAZymes had extensive connections with different species, which implied that horizontal gene transfer (HGT) might exist. One study (57) also found that the rumen microbiome could obtain CAZymes from other bacteria via HGT, which supports our assumptions.

Moreover, according to metagenomic sequencing, we further analyzed the changes in microbial metabolic function caused by MR feeding. The results showed that glycolysis and butyrate metabolism were heavily affected by MR feeding. The adequate intake of carbohydrates from the MR increased the rate of glucose production, and then glucose was converted into pyruvate. As the fermentation substrate, pyruvate might be the primary driving force for the following VFA fermentations (58). The functional enzyme genes closely related to *Prevotella* were the important executors during these processes, which showed positive correlations with butyrate.

In conclusion, we demonstrated the significant effect on the microbial composition of yak calves caused by MR feeding. In particular, the abundance of *Prevotella* members increased in the TRT group. A total of 138 ARGs conferring resistance to 17 different classes of antimicrobials in both groups were detected, meaning that the rumen microbiome plays a critical role in the natural preservation of AMR. MR feeding also drove the changes in rumen resistome, which might be related to their bacterial origins. In addition, MR feeding activated the expression of CAZyme families and functional enzymes related to the carbohydrate metabolic pathway, contributing to the degradation of cellulose. Moreover, the functional enzyme gene attached to *Prevotella* species might be the key to accelerating butyrate production. These findings suggested that in response to MR feeding, except for the changes in rumen microbial composition in yaks, the ARGs and functional genes could also be affected. Our study provided beneficial evidence for the regulation of the rumen microbiome and the intervention of AMR in ruminants by dietary or nutritional methods.

## MATERIALS AND METHODS

### Animals and sample collection

This study was conducted under the guidance of the Animal Ethics Committee of the Institute of Feed Research of the Chinese Academy of Agricultural Sciences (protocol number: AEC-CAAS-20190615; approval date: 5 June 2019). Sixty healthy female yak claves (30 days old, 22.5 ± 0. 9 kg) were chosen from a local yak farm (Qiangtang Animal Husbandry Development Co., Ltd., Nagqu, Tibet, China, N 31.48, E 92.05, altitude 4,436 m) and randomly divided into two groups. One group of calves (CON, *n* = 30) lived with their dams and consumed milk, while the other group (TRT, *n* = 30) were fed with a commercial MR (Table S1) using an artificial milk bottle. The total daily feeding of MR is 1.5% of body weight and was fed twice at 08:00 and 18:00. All dams were also fed two times daily according to the farm’s feeding management schedule, with a total mixed ration consisting of 35% corn silage, 28% peanut straw, 7% garlic straw, 3% soybean residue, 15% corn, 6% wheat bran, and 6% soybean meal. We ground the feed and passed it through a 1-mm sieve. After drying in an oven at 135°C for 2 h (method 930.15; AOAC, 1990), the dry matter content was measured. We also measured the ash content, nitrogen, neutral detergent fiber, acid detergent fiber, calcium, and total phosphorus according to the standardized method. Crude protein was calculated as 6.25 × nitrogen. During the experiment, yak calves had no access to their dam’s feed and had *ad libitum* access to water and starter (Table S2). The experiment lasted for 120 days. At the end of the experiment, eight yaks were randomly selected from each group, and their rumen fluid was collected (200 mL) from multiple locations using an 8-mm-diameter pharynx tube before morning feeding. The rumen fluid samples were kept in a liquid nitrogen tank and then immediately transferred to −80°C for subsequent analysis of the concentrations of VFAs and DNA extraction.

### Determination of rumen fermentation parameters

The rumen fluid samples were thawed at 4℃ and then centrifuged at 2,500 × *g* at room temperature. Next, 1 mL of the supernatant per sample was separated and transferred into a 1.5-mL centrifuge tube, which contained 0.2 mL of metaphosphoric acid solution (25% [wt/vol]). Then, the mixture was centrifuged at 10,000 × *g* at 4℃ after being placed in an ice water set at 4℃ for 30 min. The supernatant was collected to detect the VFA concentration using gas chromatography (GC-6800, Beijing Beifen Tianpu Instrument Technology, Co., Ltd., China).

### DNA preparation, library construction, and metagenomic sequencing

Total genomic DNA from 16 rumen fluid samples was extracted using the FastDNA Spin Kit for Soil (MP Biomedicals, 3 Hutton Center Drive, Suite 100, Santa Ana, CA, USA). A 1.0% agarose gel electrophoresis and a Nanodrop ND-1000 (Thermo Fisher Scientific, Wilmington, DE, USA) were applied to check the purity and integrity of the genomic DNA. Eligible DNA samples were randomly interrupted into fragments about 350 bp, and then the whole library was initially quantified using a Qubit 2.0 fluorimeter (Invitrogen, Carlsbad, CA, USA) and performed on the Illumina HiSeq4000 PE150 (2× 150 bp) platform at Beijing Allwegene Technology Co., Ltd. (Beijing, China).

### Metagenome assembly and bioinformatics analysis

The original off-machine data were qualified using Trimmomatic (version 0.36) (59), including removing the adapters, low-quality reads (quality scores <25), and filtering out the reads with <150 bp. The reads after quality control were compared with the host reference genome using bowtie2 software, and the reads aligned to the host were removed (60). To analyze the microbial composition, taxonomic annotation was identified using DIAMOND (version 0.8.23.85) for alignment with the NCBI nr database (61). Taxonomic classification was visualized for presentation using the softwires of MEGAN 6 (62) and Krona (63). To eliminate any bias caused by the difference in sequencing depth, the read counts within each sample were normalized into counts per million (CPM) for downstream analysis (64). In order to annotate ARGs to predict bacterial taxa, all the reads of ARGs were aligned with BLASTx against the NCBI nr database (21). To reveal the functional components of fecal microbial communities, a non-redundant gene set was annotated against the Carbohydrate-Active EnZymes database (CAZy) (65) using hmmscan (hmmer-3.1b2) (66).

### Statistical analysis

The statistical analyses were performed by R software (version 3.5.1). Alpha diversity and bacterial abundance of the two groups were compared using a two-tailed Wilcoxon signed-rank test. Beta diversity based on abundance was tested using an ANOSIM. The LEfSe (available online at galaxy.biobakery.org) was applied to identify the signature microbiota and ARGs between the CON and TRT groups. LDA score >2.5 and *P* < 0.05 were used as criteria for judging the significant effect size. Correlation analysis was tested via Spearman’s correlation, and the network was visualized using Gephi V0.9.2 and Cytoscape V3.5.1.

## Data Availability

Rumen metagenome sequences were deposited into the NCBI Sequence Read Archive (SRA) under BioProject number PRJNA806696.

## SUPPLEMENTAL MATERIAL

Table S1, Table S2, Figures S1 to S3 spectrum.01314-23-s0001.docx Supplemental results.

ASM does not own the copyrights to Supplemental Material that may be linked to, or accessed through, an article. The authors have granted ASM a non-exclusive, world-wide license to publish the Supplemental Material files. Please contact the corresponding author directly for reuse.
