# Supplementary material for: Altered microbiota, antimicrobial resistance genes, and functional enzyme profiles in the rumen of yak calves fed with milk replacer
Source: Microbiol Spectr. 2023 Nov 28;12(1):e01314-23. doi: 10.1128/spectrum.01314-23 (PMC10871699; doi:10.1128/spectrum.01314-23)
Supplement: Table S1, Table S2, Figures S1 to S3 — Supplemental results. [file spectrum.01314-23-s0001.docx]

*Supplementary Information*

**Altered microbiota, antimicrobial resistant gene and functional enzymes profiles in the rumen of yak calves fed with milk replacer**

**Yimin** **Zhuang, ^a^ Wei Guo, ^b^** **Kai Cui, ^a^** **Yan Tu, ^a^** **Qiyu Diao, ^a^ Naifeng Zhang, ^a^****^*^ Yanliang Bi, ^a^****^*^ Tao Ma ^a*^**

^a^Key laboratory of Feed Biotechnology of the Ministry of Agriculture and Rural Affairs, Institute of Feed Research, Chinese Academy of Agricultural Sciences, Beijing 100081, China

^b^Key Laboratory of Animal Genetics, Breeding and Reproduction in the Plateau Mountainous Region, Ministry of Education, Guizhou University, Guiyang, China

^*^ Corresponding Author: Naifeng Zhang, email: zhangnaifeng@caas.cn; Yanliang Bi, email: biyanliang@caas.cn; Tao Ma, email: [matao@caas.cn](mailto:matao@caas.cn)

**Table S1.** Nutritional components of milk replacer

**Table S2.** Nutritional components of starter

**Figure S1.** Microbial composition at phyla level in response to MR feeding

**Figure S2.** Microbial composition at genus level in response to MR feeding

**Figure S3.** The network reveals the co-occurrence patterns between species and CAZymes

| **Items** | **Milk Replacer** |
| --- | --- |
| Chemical composition, % of DM basis | |
| DM (%) | 94.7 |
| CP (%) | 22.9 |
| EE (%) | 16.0 |
| NDF (%) | --- |
| ADF (%) | --- |
| Ash (%) | 4.3 |
| Ca (%) | 0.9 |
| P (%) | 0.5 |

**Table S1.** Nutritional components of milk replacer

DM: Dry matter; CP: Crude protein; EE: Ether extract; NDF: Neutral detergent fiber;

ADF: Acid detergent fiber; Ash: Crude ash

**Table S2.** Nutritional components of starter

| **Items** | **Starter Pellets** |
| --- | --- |
| Chemical composition, % of DM basis | |
| DM (%) | 92.9 |
| CP (%) | 19.40 |
| EE (%) | 3.73 |
| NDF (%) | 33.23 |
| ADF (%) | 15.93 |
| Ash (%) | 7.99 |
| Ca (%) | 0.95 |
| P (%) | 0.70 |

DM: Dry matter; CP: Crude protein; EE: Ether extract; NDF: Neutral detergent fiber;

ADF: Acid detergent fiber; Ash: Crude ash


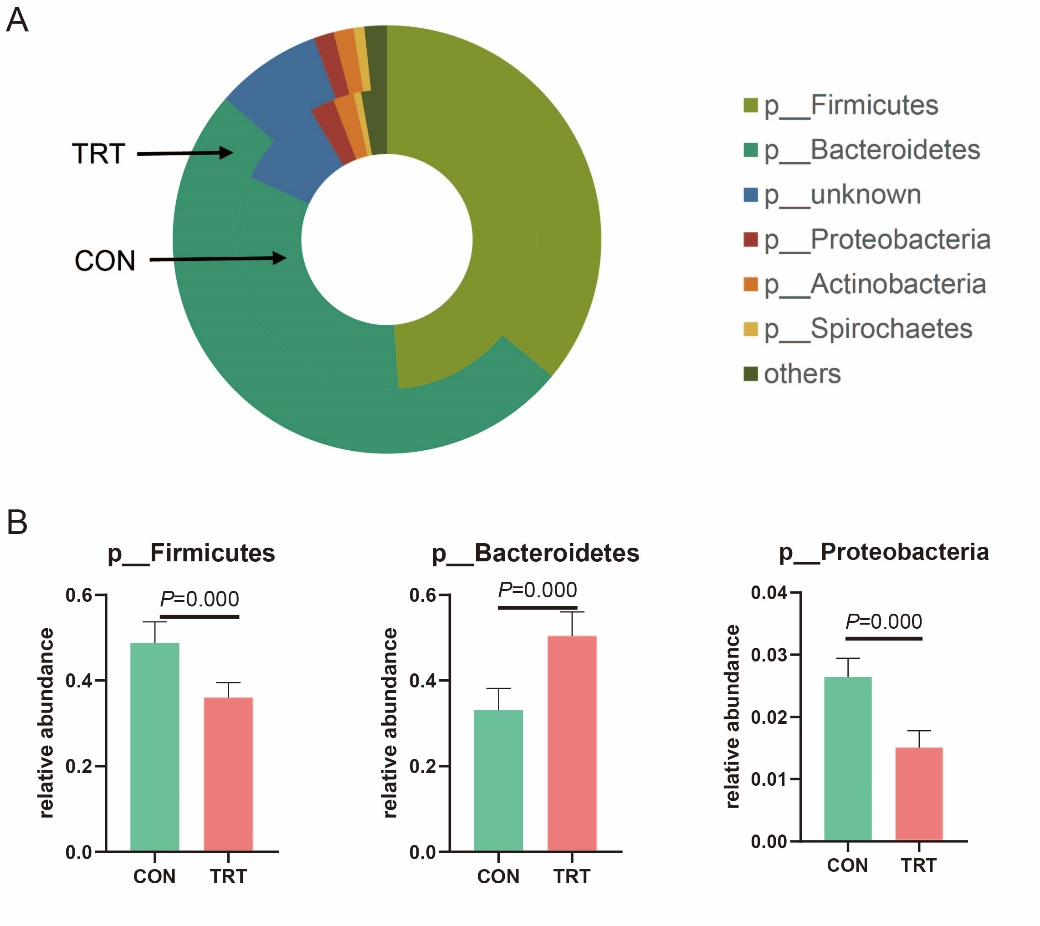


**Figure S1.** Microbial composition at phyla level in response to MR feeding. **A** The top 7 bacterial phyla were selected. The doughnut shows the changes from CON to TRT group. The inner doughnut represents the CON group, and the outer doughnut represents the TRT group. **B** The bar charts demonstrate the phyla which were affected significantly by MR feeding. P value＜0.05 in each block means the significant differences in the abundance of bacteria between CON and TRT group.


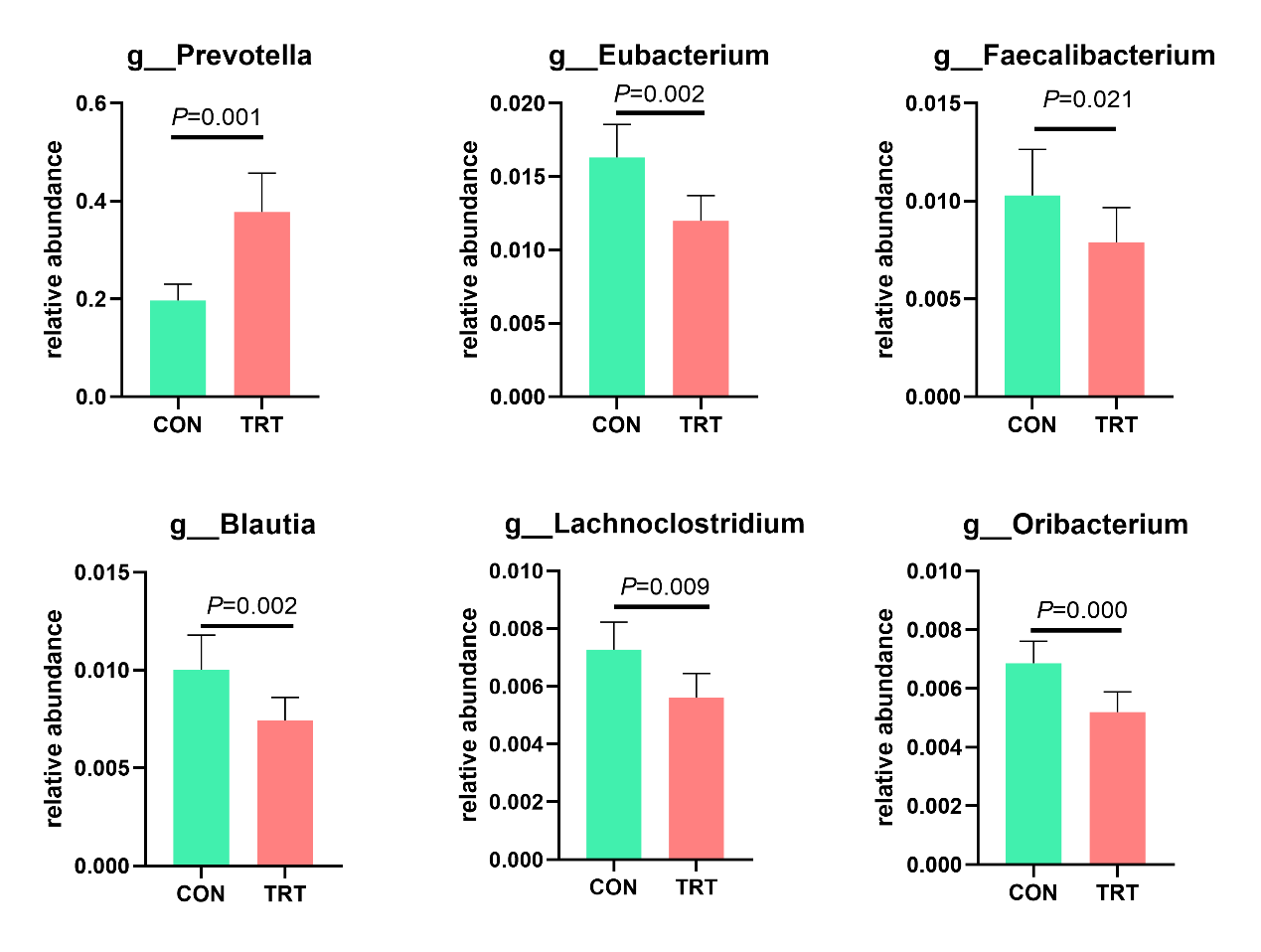


**Figure S2.** Microbial composition at genus level in response to MR feeding. The bar charts demonstrate the genera which were affected significantly by MR feeding. P value＜0.05 in each block means the significant differences in the abundance of bacteria between CON and TRT group.


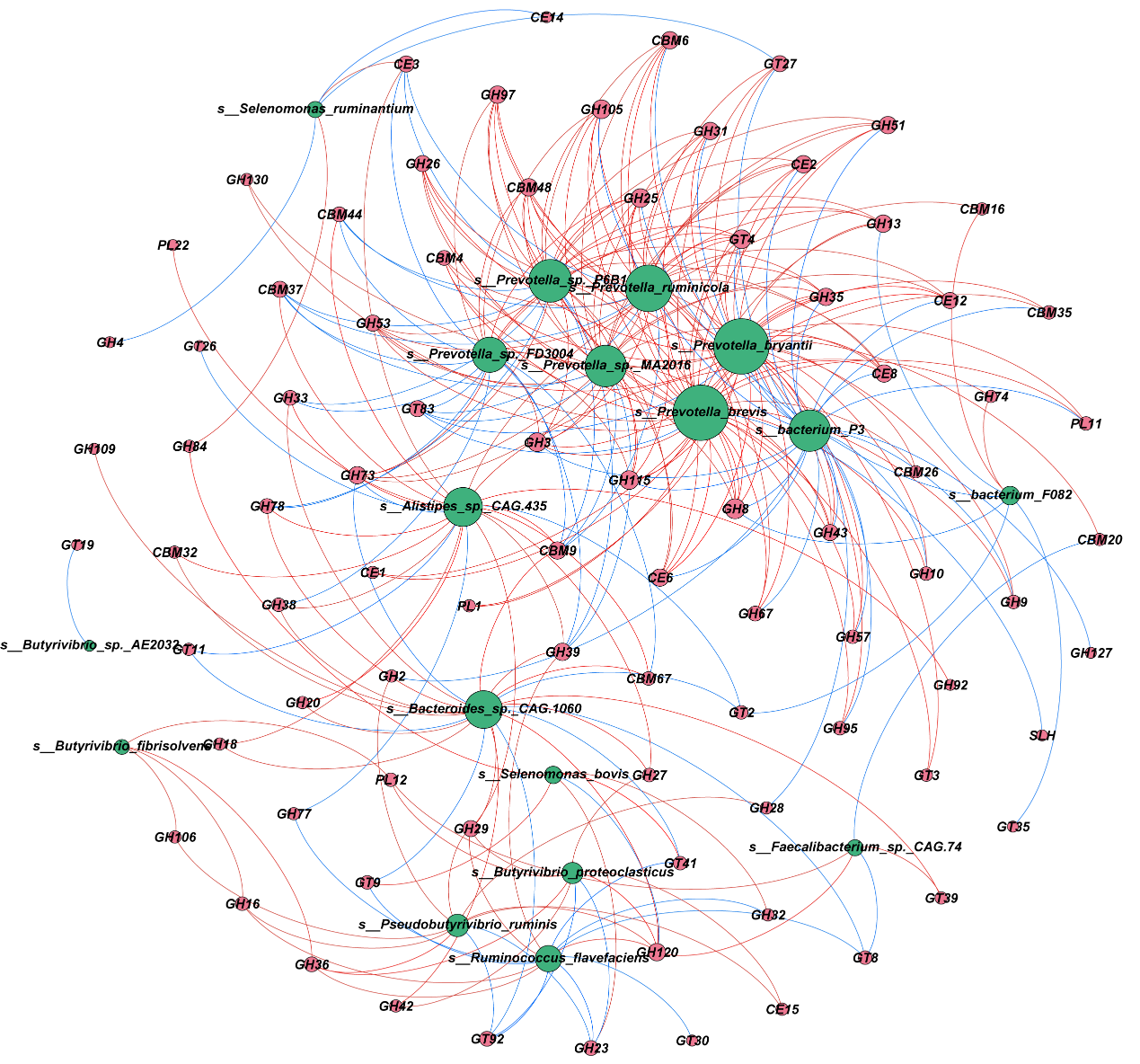


**Figure S3.** The network reveals the co-occurrence patterns between species and CAZymes. Different nodes represent different species or CAZyme. The size of nodes depends on the number of connections (degree). The color of nodes is corresponding to species (green) and CAZyme (red). The color of the line represented the positive (red) or negative correlation (blue). Only significant connections were shown in the network (*P* < 0.05, |r| > 0.5).
